# Milling-Induced Chitosan–HPMC Microstructural Organisation Determines the Stability Window of Concentrated Azelaic Acid Nanosuspensions

**DOI:** 10.3390/pharmaceutics18091071

**Published:** 2026-08-27

**Authors:** Sandra Miočić, Andrea Rašić, Jelena Torić, Michela Abrami, Kristina Ferderber, Biserka Cetina-Čižmek, Mario Grassi, Jelena Filipović-Grčić

**Affiliations:** 1 PLIVA Croatia Ltd., 10000 Zagreb, Croatia; sandra.miocic@pliva.com (S.M.); andrea.rasic@pliva.com (A.R.); jelena.toric@pliva.com (J.T.); kristina.ferderber@pliva.com (K.F.); biserka.cetina-cizmek@pliva.com (B.C.-Č.); 2 University of Zagreb Faculty of Pharmacy and Biochemistry, 10000 Zagreb, Croatia; 3 Department of Engineering and Architecture, University of Trieste, I-34127 Trieste, Italy; michela.abrami@dia.units.it (M.A.); mario.grassi@dia.units.it (M.G.)

**Keywords:** azelaic acid, concentrated nanosuspensions, wet media milling, HPMC–chitosan stabilisation, rheology

## Abstract

**Objectives:** The physical stability of concentrated azelaic acid nanosuspensions cannot be predicted solely from interfacial stabilisation but reflects the interplay between particle size-dependent thermodynamic driving forces, polymer-mediated rheological structuring and the resulting bulk microstructural organisation. This study employed an A-optimal design of experiments (18 runs) to investigate formulation–process relationships in concentrated azelaic acid nanosuspensions (10–20% *w*/*w*) stabilised with a dual HPMC–chitosan system. **Methods:** Particle size, ζ-potential, rheological behaviour, solid-state properties and in vitro permeation across Strat-M® membranes were evaluated, and formulation–process relationships were analysed using multivariate modelling and logistic regression. **Results:** Particle size (342–1118 nm; R^2^ = 0.97) was primarily governed by the applied milling regime. Logistic regression demonstrated a size-dependent probability of crystal growth, with the estimated particle-size transition point (predicted probability = 0.5) shifting from approximately 424 nm after preparation to 594 nm following accelerated storage at 40 °C. CHI concentration controlled ζ-potential (+18.5 to +47.9 mV), although ζ-potential alone did not adequately explain the observed storage stability. Where measurable, zero-shear viscosity (68–45,462 mPa·s) reflected substantial differences in low-shear rheological structuring, while formulations containing higher HPMC concentrations generally exhibited improved stability, consistent with polymer-mediated kinetic constraints on crystal growth. In vitro permeation studies using Strat-M^®^ membranes demonstrated permeation behaviour consistent with structured diffusion-controlled systems, exhibiting lower flux but more uniform permeation profiles than the reference formulation under the applied experimental conditions. **Conclusions:** Integration of multivariate modelling with rheological, solid-state and permeation characterisation provided an integrated understanding of the formulation–process relationships governing the short-term physical stability and comparative in vitro transport behaviour of concentrated dermal AZA nanosuspensions.

## 1. Introduction

Nanosuspensions are colloidal dispersions of drug nanocrystals stabilised by surfactants or polymers. Nanonisation increases surface area and dissolution rate, but the associated increase in surface free energy may compromise physical stability, particularly at high solid contents [1,2,3,4,5].

In dermal delivery, nanosuspensions can maintain high thermodynamic activity at the skin surface, promoting passive diffusion across the stratum corneum [6,7,8]. Particle sizes of 300–700 nm may further favour follicular accumulation and local depot formation within the pilosebaceous unit, supporting sustained local drug availability [9,10]. These systems are therefore attractive for topical therapy, particularly where sustained local exposure rather than systemic absorption is desired [11].

The physical stability of nanosuspensions is determined by interfacial stabilisation and bulk interactions. Although the adsorption of stabilisers provides electrostatic, steric, or electrosteric repulsion, stability cannot be predicted solely from ζ-potential. Instead, it reflects the combined effects of interfacial adsorption, drug physicochemical properties, solid fraction, and process-induced stresses [4,11,12]. At high solid contents, electrostatic repulsion alone is often insufficient, and steric hindrance, together with kinetic constraints on particle mobility, becomes critical.

Drug solubility plays a key role in nanosuspension stability. For medium soluble compounds (1–33 mg/mL), rapid partial dissolution of nanocrystals may induce transient supersaturation, promoting Ostwald ripening and crystal growth, particularly at higher solid fractions and temperatures [7,12,13]. This behaviour differs from that of poorly soluble drugs and introduces additional challenges in concentrated systems, where dissolution–recrystallisation dynamics compete with kinetic stabilisation mechanisms [3,14].

Azelaic acid (AZA), a medium soluble dicarboxylic acid with established dermatological use, exemplifies these challenges. Clinically relevant topical formulations require high drug loadings (15–20%) [15], whereas most reported AZA nanosuspensions contain substantially lower solid contents and rely on post-processing steps to achieve therapeutic concentrations [16,17]. These approaches increase manufacturing complexity and may compromise nanocrystal integrity.

Directly processable high solid-content nanosuspensions (>10% *w*/*w*) therefore represent an attractive alternative. However, increasing the dispersed phase fraction intensifies particle–particle interactions, alters rheological behaviour, and enhances thermodynamic driving forces for crystal growth [18,19]. In our previous work, single-stabiliser systems did not provide sufficient milling operability or stability, while HPMC alone caused an excessive viscosity increase during milling [20]. In contrast, a combined HPMC–chitosan (CHI) system enabled processing up to 20% *w*/*w* AZA, although storage stability remained limited at higher loadings. The mechanistic interplay between interfacial charge, polymer-mediated structuring, and process energy is still insufficiently understood.

A systematic framework is therefore needed to understand how interfacial stabilisation and rheology-derived bulk microstructural organisation together influence the physical stability of concentrated nanosuspensions. Based on our previous observations [20], which showed that neither HPMC nor chitosan alone provided sufficient processability and stability at AZA loadings above 10%, we hypothesised that their combination would provide a rheologically structured stabilisation system capable of maintaining both milling operability and resistance to storage-induced crystal growth. Using a Design of Experiments (DoE) approach [21], this study investigates how formulation composition and milling conditions define the stability window of concentrated AZA nanosuspensions through their effects on particle size, interfacial stabilisation, rheological behaviour, and in vitro transport behaviour.

## 2. Materials and Methods

### 2.1. Materials

Azelaic acid (Dermaz99^®^, BASF (Ludwigshafen, Germany)). with a D90 particle size of approximately 10 µm was supplied by BASF (Ludwigshafen, Germany). Hydroxypropyl methylcellulose (HPMC, Methocel^®^ E5 LV) was obtained from DuPont (Wilmington, NC, USA), and low-molecular-weight chitosan (approximate molecular weight 150–250 kDa, based on the manufacturer’s viscosity classification; viscosity 20–200 mPa·s for a 1% solution in acetic acid at 20 °C; degree of deacetylation ≥75%) was purchased from Sigma-Aldrich (Steinheim, Germany); both were used as stabilisers. Acetic acid and sodium hydroxide (Merck, Darmstadt, Germany) were used for pH adjustment, and purified water served as the dispersion medium.

For the wet milling process, yttria-stabilised zirconium oxide beads (Silibeads^®^ type ZY-P Pharma, diameter 0.1–0.2 mm) were purchased from Sigmund Linder GmbH (Warmensteinach, Germany). Polystyrene milling beads (PuroMill^®^ PM5000, diameter 0.45–0.55 mm) were supplied by Purolite (King of Prussia, PA, USA). All other chemicals and reagents were of analytical grade.

### 2.2. Preparation of AZA Nanosuspensions

AZA-NS were prepared by wet media milling (WMM) using a Dyno-Mill^®^ Researchlab agitator bead mill (Willy A. Bachofen AG, Muttenz, Switzerland). Chitosan (CHI) was dissolved in 0.1 N acetic acid, and hydroxypropyl methylcellulose (HPMC) in purified water. AZA (10–20% *w*/*w*) was dispersed into the combined stabiliser solutions to form a coarse suspension, which was stirred for 10 min at 600 rpm. The suspension was pre-homogenised at 3500 rpm for 10 min using a high-shear homogeniser (Silverson LM5, Silverson Machines Ltd., Chesham, UK), followed by pH adjustment to 4.5 using 1 N NaOH. Subsequently, 150 g of the suspension was processed at 4000 rpm. Two bead types were used: yttria-stabilised zirconia (YZB; 0.1–0.2 mm) and polystyrene beads (PB; 0.45–0.55 mm). Based on preliminary process optimisation, fixed milling times of 60 min for YZB and 240 min for PB were applied to all formulations in the respective bead category to achieve efficient particle-size reduction under each milling regime. Milling was performed in a closed-loop system with 55 mL beads (~70% chamber volume) under continuous cooling. Under these conditions, the suspension temperature typically reached 28–32 °C during YZB milling and 23–25 °C during PB milling, with the highest temperatures observed for formulations containing 20% *w*/*w* AZA. All concentrations are expressed as % *w*/*w* relative to the total formulation mass.

### 2.3. Formulation–Process Optimisation Using Design of Experiments

A Quality-by-Design (QbD) framework was applied to investigate the formulation–process space for concentrated AZA-NS. The quality target product profile (QTPP) specified a physically robust, high solid-content nanosuspension with controlled particle size distribution and appropriate rheological properties. Accordingly, CQAs were defined as Z-average particle size, polydispersity index (PDI), ζ-potential, rheological parameters (infinite-shear viscosity, *η_∞_*, and flow behaviour index, *n*), and short-term stability.

Critical material attributes (CMAs) and critical process parameters (CPPs) were identified based on previous stabiliser screening [20] and preliminary experiments. AZA, HPMC, and CHI concentrations were selected as formulation variables, while the wet media milling regime was included as a categorical process factor. Two practically relevant wet media milling regimes established during preliminary process optimisation were investigated: yttria-stabilised zirconia (YZB) beads (0.1–0.2 mm; 60 min milling) and polystyrene (PB) beads (0.45–0.55 mm; 240 min milling) (Table 1).

An A-optimal partial factorial design was generated using JMP^®^ Pro 17.2 (SAS Institute Inc., Cary, NC, USA). Formulation variables were studied at three levels (–1, 0, +1): AZA (10–20% *w*/*w*), HPMC (1.5–3.0% *w*/*w*), and CHI (0.1–0.3% *w*/*w*), while the two milling regimes were evaluated as a categorical factor (Table 1). The design comprised 18 experimental runs and allowed estimation of main effects, two-factor interactions, and selected quadratic terms. Each experimental run corresponded to an independently prepared nanosuspension batch. For each experimental run, three DLS measurements and duplicate rheological measurements were performed on the corresponding batch. These represent analytical replicates and therefore quantify analytical repeatability rather than between-batch variability. The design included two pairs of independently prepared replicate batches at identical design points (DOE-2/DOE-3 and DOE-7/DOE-11). DOE-18 represented the centre of the continuous formulation variables under the PB milling regime and was therefore not an identical replicate of the YZB centre-point formulations. Milling speed and bead volume fraction were kept constant across all experiments, whereas bead type, bead size and milling duration defined the two investigated milling regimes.

### 2.4. Short-Term Physical Stability Study

During the short-term physical stability study of AZA nanosuspensions, susceptibility to crystal growth and aggregation under ambient and accelerated conditions was evaluated. Freshly prepared samples were transferred into sealed glass vials and stored at room temperature (RT) and at 40 °C for 10 days. Samples were analysed on day 0 and day 10 for changes in particle size distribution and polydispersity index (PDI) using dynamic light scattering (Section 2.5.1). Optical microscopy was also used to detect microscopically visible structures not captured by DLS. Crystal growth was recorded as a binary response (present = 1, absent = 0) based on the presence or absence of needle-like crystalline structures and was used for logistic regression modelling within the DoE framework to evaluate formulation robustness.

### 2.5. Characterisation of AZA Nanosuspensions

The physicochemical properties of AZA nanosuspensions were characterised in terms of particle size distribution, ζ-potential, morphology, and rheological behaviour using the analytical techniques described below.

#### 2.5.1. Particle Size, Polydispersity Index, and ζ-Potential

Particle size distribution and ζ-potential were determined by dynamic light scattering (DLS) using a Zetasizer Nano ZS (Malvern Instruments, Malvern, UK). Particle size was expressed as the intensity-weighted mean diameter (Z-average), and the polydispersity index (PDI) described the distribution width. Samples were diluted with saturated AZA solution, which had been filtered through a 0.1 μm membrane, to minimise multiple scattering and prevent dissolution. Measurements were performed at 25 °C (173° backscattering angle), and values are reported as the mean of three measurements. Morphology and the presence of needle-like crystalline structures were assessed by optical microscopy (Olympus BX51, Olympus, Tokyo, Japan) at 200× and 500× magnification, with images recorded using a digital camera.

#### 2.5.2. Rheological Evaluation

The rheological behaviour of AZA nanosuspensions was determined using a rotational rheometer (Anton Paar MCR 302, Anton Paar GmbH, Graz, Austria) equipped with a Peltier temperature control unit and a cone–plate geometry (CP25; 1°, 25 mm, gap 0.049 mm). Measurements were performed in duplicate at 25 °C. Flow curves were obtained over a shear rate range of 0.1–10,000 s^−1^ to capture both low- and high-shear behaviour relevant to structural stability, processing, and application.

Experimental flow curves were fitted to the Cross model [22]:

$$\eta (\dot{\gamma })=\eta_{\infty }+\frac{\eta_{0}-\eta_{\infty }}{1+(\lambda \dot{\gamma }{)}^{n}}$$

where *η_0_* is the zero-shear viscosity, *η∞* the infinite-shear viscosity, *λ* the characteristic relaxation time constant, and *n* the flow behaviour index. Model parameters were obtained by nonlinear regression.

#### 2.5.3. X-Ray Diffraction (XRD)

For XRD analysis, AZA nanosuspensions were poured as a thin layer into aluminium sample holders and dried at 40 °C for 24 h. The dried samples were subsequently homogenised in a mortar before analysis. X-ray diffraction analysis was carried out using an X’Pert Pro diffractometer (PANalytical, Almelo, The Netherlands) equipped with a monochromator providing CuKα radiation. Diffraction patterns were recorded over a 2θ range of 3° to 40°, with a step size of 0.017° and a counting time of 102 s per step.

#### 2.5.4. Scanning Electron Microscopy (SEM)

The morphology of AZA nanocrystals was examined using scanning electron microscopy (JSM-5800, JEOL Ltd., Akishima, Tokyo, Japan). Samples were diluted, lyophilised according to a previously established protocol [23] and mounted on aluminium stubs with conductive carbon tape. Before imaging, samples were sputter-coated with a thin gold layer. Images were acquired and processed using Aztec software (Oxford Instruments NanoAnalysis, High Wycombe, UK).

#### 2.5.5. In Vitro Permeation Across Strat-M Membrane

In vitro permeation studies were conducted using an automated Franz diffusion cell system (Phoenix™ RDS, Teledyne Hanson, Chatsworth, CA, USA) comprising six vertical diffusion cells (15 mL receptor volume) maintained at 32 °C with continuous stirring at 500 rpm. The receptor compartment was filled with 1 M acetate buffer (pH 5.5), chosen on the basis of its experimentally determined AZA solubility of 19.9 mg/mL at 32 °C. A Strat-M^®^ membrane (Merck KGaA, Darmstadt, Germany) with an exposed diffusion area of 1.77 cm^2^ was mounted between the donor and receptor compartments, with the shiny side facing the donor compartment. Formulations (1 mL) were applied to the donor compartment. Samples (200 µL) were withdrawn at 2, 4, 6, 8, 12, 16, 20, 24, 32, 38, 44, 48, 54, 62, 68 and 72 h, with immediate replacement by an equal volume of fresh receptor medium. Cumulative permeated amounts were corrected for sample withdrawal and receptor medium replacement. AZA concentrations in receptor samples were quantified by a validated HPLC method [16] using an Agilent 1260 Infinity II system equipped with a Kinetex C18 column (Agilent Technologies, Inc., Santa Clara, CA, USA). The mobile phase consisted of phosphate buffer (pH 2.0) and acetonitrile (75:25, *v*/*v*) at a flow rate of 1.2 mL/min, with UV detection at 210 nm. Experiments were performed in triplicate (n = 3). Flux (J) was determined from the linear portion of the cumulative permeation profiles, and the apparent permeability coefficient (P_app_) was calculated as J/C_d_, where C_d_ is the nominal total AZA concentration in the donor formulation. Accordingly, P_app_ should be interpreted as an apparent comparative permeability parameter rather than an intrinsic membrane permeability coefficient.

### 2.6. Statistical Analysis and Regression Modelling

Statistical analysis was conducted using JMP^®^ Pro 17.2 (SAS Institute Inc., Cary, NC, USA). Multiple linear regression models were used to evaluate main effects and two-factor interactions. Model adequacy was assessed by analysis of variance (ANOVA), adjusted coefficient of determination (R^2^adj), lack-of-fit testing, and residual diagnostics. Residual normality was evaluated using normal probability plots and the Shapiro–Wilk test, while variance homogeneity was assessed by visual inspection of residual-versus-predicted plots. No major deviations from residual normality or homoscedasticity were observed for the evaluated response models, and no response transformations were required. Replicated centre-point formulations were included in the A-optimal design to support the estimation of pure error and lack-of-fit assessment. Binary stability outcomes were analysed using nominal logistic regression. Rheological flow curves were fitted to the Cross model by nonlinear regression, and the statistical significance of the fitted models was evaluated using the F-test. Correlations between rheological parameters and formulation variables were evaluated using Spearman’s rank correlation. Statistical significance was set at *p* < 0.05.

## 3. Results and Discussion

In our previous work, we established the mechanistic basis for stabilising concentrated azelaic acid (AZA) nanosuspensions prepared by wet media milling, demonstrating that a combined HPMC–CHI system provides robust electrosteric stabilisation without altering the crystalline structure of AZA at concentrations of up to 10% *w*/*w* [20].

While nanosuspensions are predominantly reported for poorly soluble drugs at low solid loadings, systematic investigations of concentrated systems containing moderately soluble compounds remain limited. AZA, with moderate aqueous solubility (~2.4 mg/mL at 20 °C), represents a challenging model, as physical stability at high solid contents is strongly influenced by temperature-dependent solubility and crystal growth.

Building on this framework, the present study evaluates how formulation composition and milling parameters influence the CQAs of concentrated AZA nanosuspensions using a multivariate DoE approach. AZA concentration (10–20% *w*/*w*), HPMC concentration (1.5–3.0% *w*/*w*), CHI concentration (0.1–0.3% *w*/*w*), and milling regime (implemented using YZB or PB) were investigated as independent variables, while Z-average, PDI, ζ-potential, rheological parameters, and crystal growth were assessed as responses. All formulations were successfully prepared at pH 4.5 ± 0.1, and regression modelling was used to identify significant formulation–process relationships (Table 2 and Table 3).

The results are presented, covering particle size control, electrokinetic stabilisation, rheological behaviour, and short-term physical stability, which define the operational boundaries for processability and robustness of concentrated AZA nanosuspensions.

### 3.1. Milling Efficiency and Particle Size Distribution

Milling efficiency was assessed using particle size distribution metrics (Z-average and PDI) across the formulation–process space (Table 2). Z-average values ranged from 342.7 ± 2.6 nm to 1118.0 ± 18.0 nm, while PDI ranged from 0.186 ± 0.016 to 0.393 ± 0.009.

Clear differences between milling media were observed. YZB produced smaller particles (342–624 nm) with generally lower PDI values (0.186–0.344), whereas PB produced larger particles (446–1118 nm) and higher PDI values (0.235–0.393), reflecting more efficient energy transfer and higher stress intensity during milling with YZB [24,25].

**Figure 1:**
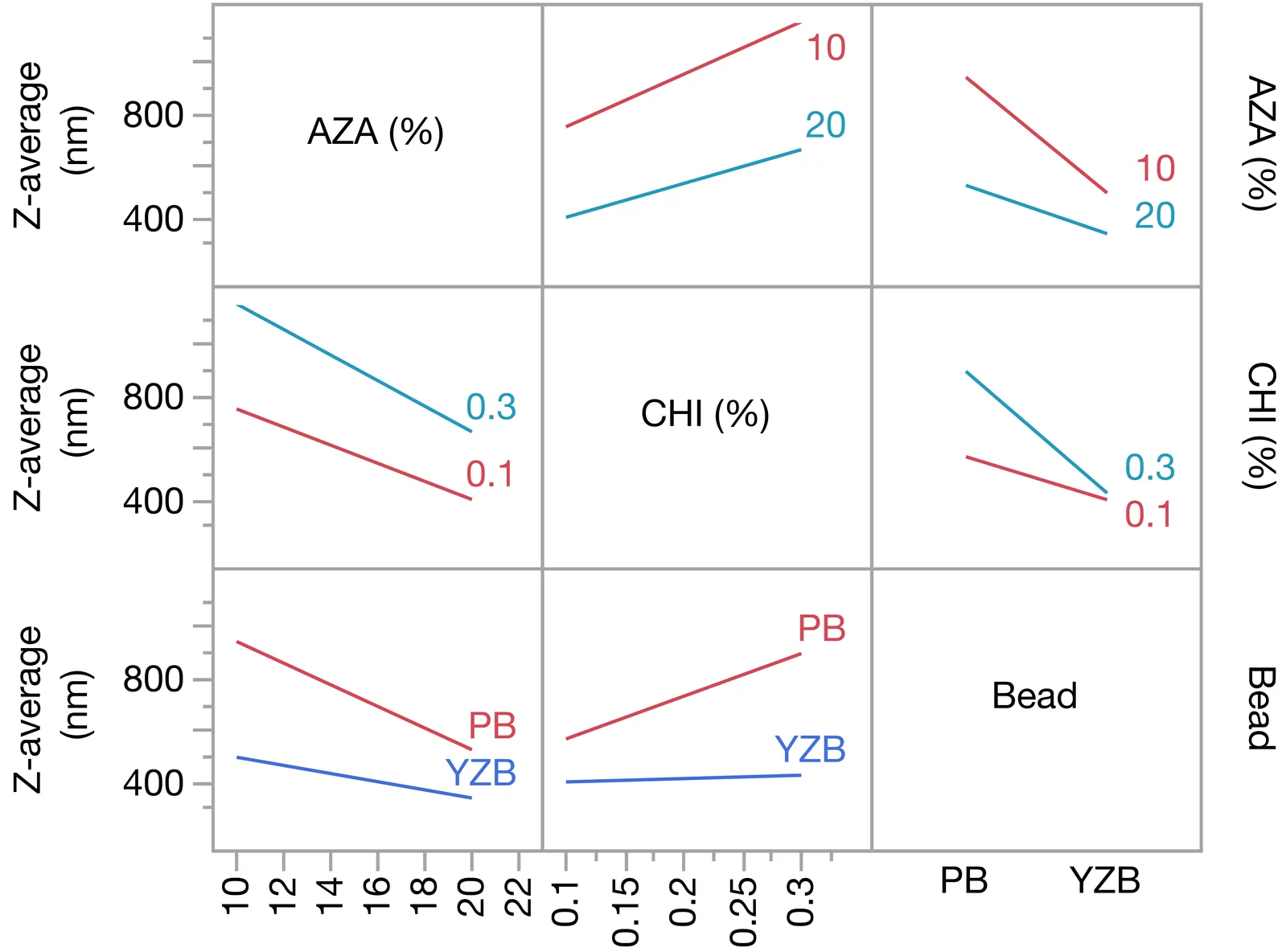
Interaction profiles illustrating the effects of AZA concentration, CHI concentration, and milling regime on particle size (Z-average) within the investigated formulation–process space.

Regression analysis confirmed that particle size was primarily associated with the investigated milling regime (R^2^ = 0.97), with significant interactions between bead regime and formulation variables, particularly AZA% × Bead and CHI% × Bead (Table 3). These interactions indicate that the effects of drug loading and polymer concentration on particle breakage depend on the applied stress regime, reflecting a coupled formulation–process response [24]. Interaction plots (Figure 1) further illustrate this behaviour: increasing AZA concentration results in a more pronounced decrease in particle size under PB conditions, while the YZB milling regime exhibits a more moderate response. A similar trend is observed for CHI concentration. Although the model captured the principal formulation–process trends, the statistically significant lack of fit for Z-average indicates that additional non-linear contributions were not fully described by the reduced regression model, likely reflecting complex stress–microstructure interactions at high solid loading [19,26]. Accordingly, the model should be interpreted as describing the principal formulation–process relationships within the investigated formulation–process space rather than as a precise predictive model.

Analysis of PDI confirmed that AZA concentration and milling regime significantly influenced distribution width (Table 3). Increasing AZA concentration resulted in lower PDI values, consistent with enhanced particle–particle interactions and more uniform breakage at higher solid loadings [18,27]. The YZB milling regime consistently exhibited lower PDI than PB, indicating more uniform stress distribution and improved dispersion homogeneity.

Overall, these findings demonstrate that particle breakage in concentrated AZA nanosuspensions is determined by the interplay between solid loading, polymer-mediated structuring, and stress intensity, requiring simultaneous optimisation of formulation and process conditions [26,28].

### 3.2. Interfacial Stabilisation and the Role of Chitosan

Particle size reduction during wet media milling generates high-energy crystal surfaces that require interfacial stabilisation, governed by the electrostatic and steric environment of the dispersion medium [29,30]. Across the investigated formulation-process space, ζ-potential values ranged from +18.5 mV to +47.9 mV (Table 2), indicating that all nanosuspensions had a net positive surface charge, consistent with the adsorption of cationic chitosan onto AZA nanocrystal surfaces [31,32].

The regression model for ζ-potential (Table 3) showed good predictive performance (R^2^ = 0.84, *p* < 0.0001) with no statistically significant lack of fit (*p* = 0.3164). CHI concentration had the strongest positive effect on ζ-potential (*p* < 0.0001), consistent with its protonated state at pH 4.5. In contrast, increasing AZA concentration was associated with a reduction in ζ-potential (*p* = 0.0010), suggesting decreased surface charge density or altered interfacial charge distribution at higher solid loadings.

The opposing effects of CHI and AZA concentration on ζ-potential highlight the delicate balance between electrostatic repulsion and surface saturation in concentrated systems [33,34]. At higher solid loadings, the relative availability of stabiliser per unit surface area decreases, which may reduce charge density despite increased polymer concentration [35]. These findings indicate that electrostatic stabilisation in concentrated AZA nanosuspensions depends not only on the absolute polymer content but also on its ratio to the newly generated surface area during milling.

Milling regime did not significantly affect ζ-potential (*p* = 0.3759), indicating that electrokinetic properties were governed primarily by formulation composition rather than by applied stress intensity.

### 3.3. Rheological Behaviour and Bulk Microstructural Organisation

**Figure 2:**
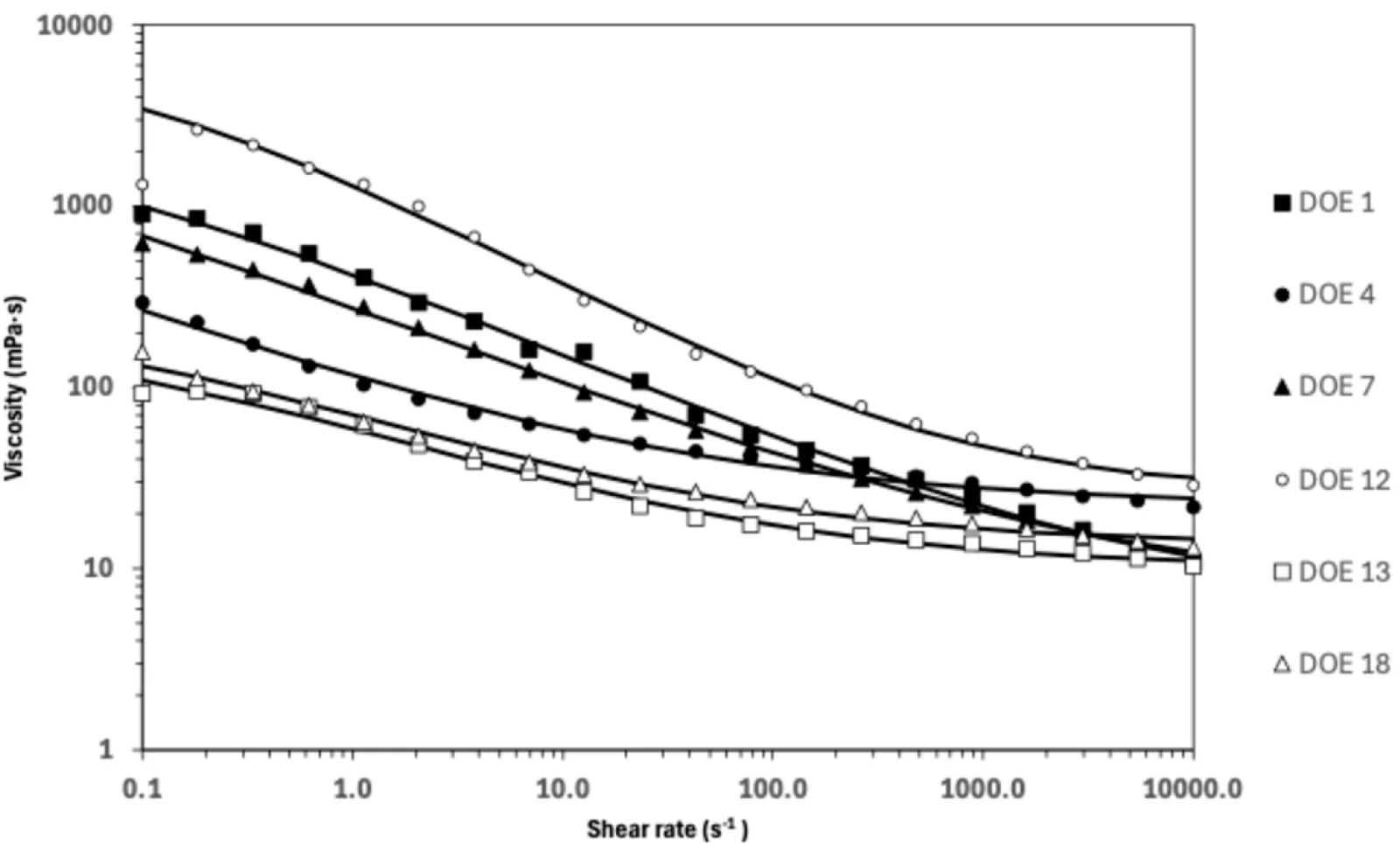
Representative flow curves of selected AZA nanosuspensions within the investigated formulation–process space. Filled markers indicate formulations processed with the YZB milling regime, while open markers represent PB-milled systems. Solid lines correspond to Cross model fitting. All systems exhibit shear-thinning behaviour consistent with structured dispersions.

While interfacial stabilisation controls surface charge development, the rheological behaviour of concentrated nanosuspensions provides indirect insight into their bulk microstructural organisation by describing how mechanical stress is transmitted, dissipated and accommodated within the dispersion. Representative flow curves for selected formulations are shown in Figure 2, and parameters obtained from Cross model fitting are summarised in Table 2.

All investigated nanosuspensions exhibited pronounced shear-thinning behaviour, consistent with the presence of rheologically structured concentrated dispersions in which polymer dynamics and dispersed nanocrystals jointly govern the overall rheological response [36].

The infinite-shear viscosity *(η_∞_*), representing the high-shear plateau of the Cross model where the rheological structure is progressively disrupted, was successfully modelled using a quadratic interaction framework (R^2^ = 0.98, *p* < 0.0001; no statistically significant lack of fit). Among the variables investigated, HPMC concentration had the strongest positive effect on *η_∞_* (*p* < 0.0001), followed by AZA concentration (*p* < 0.0001) and CHI concentration (*p* < 0.0001). Significant second-order and interaction terms, particularly AZA%^2^ and CHI%^2^, further indicate non-linear thickening at higher solid and polymer loadings.

These results suggest that high-shear viscosity in concentrated AZA nanosuspensions is determined by the combined effects of continuous-phase polymer thickening (HPMC) and an increase in the effective dispersed-phase volume fraction (*ϕ_eff_*) at elevated solid loadings, resulting from particle crowding, solvation, and possible aggregation phenomena [37]. Additionally, polymer–particle interactions may further contribute to rheological structuring, consistent with observations in HPMC-based nanocomposite systems [38].

In contrast, the flow behaviour index (*n*) showed a statistically significant but moderate dependence on AZA concentration (*p* = 0.0008) and the AZA% × CHI% interaction (*p* = 0.0212), with no statistically significant lack of fit. Increasing AZA concentration resulted in higher n values, indicating a reduced extent of shear-induced structural breakdown at higher solid loadings. This behaviour is consistent with progressive crowding and constrained rheological rearrangement within increasingly dense dispersions, as commonly observed in concentrated nanosuspensions and particle-filled polymer systems [38,39]. The significant AZA% × CHI% interaction further indicates that the effect of solid loading on flow behaviour depends on the degree of polymer-mediated structuring within the continuous phase. The Cross time constant (λ), which represents the shear-rate range over which structural transition occurs, is also reported in Table 2.

Although the Cross model adequately described the flow behaviour of most formulations, reliable estimation of zero-shear viscosity (*η_0_*) was not possible for all samples due to the absence of a clearly defined low-shear plateau. In systems where *η_0_* could be determined, values ranged from 68 mPa·s (DOE-16) to 45,462 mPa·s (DOE-2), spanning nearly three orders of magnitude and indicating substantial differences in low-shear structuring. The independently prepared replicate batches provided additional insight into the robustness of the rheological parameters. While replicate formulations showed relatively small differences in Z-average (approximately 3.5–4.2%), greater variability was observed in the low-shear Cross-model parameters. In particular, DOE-2 and DOE-3 exhibited comparable *η∞* and *n* values but markedly different fitted *η_0_* and *λ* values, whereas *η_0_* could not be reliably estimated for DOE-11 because a well-defined low-shear plateau was absent. These observations indicate that *η_0_* and *λ* are particularly sensitive to subtle batch-dependent differences in low-shear microstructure and to the identifiability of the Cross model parameters. Accordingly, *η_0_* was interpreted descriptively and was not included as a response in the DoE regression models.

Most formulations lacking a measurable zero-shear plateau contained lower CHI concentrations (0.1–0.2% *w*/*w*), suggesting that insufficient polymer-mediated structuring limited the development of a well-defined low-shear rheological structure. In contrast, systems with 0.3% CHI processed with YZB beads more frequently exhibited defined low-shear behaviour, indicating that a threshold CHI concentration may be required to promote bulk rheological structuring. This observation is consistent with reports that chitosan can promote network formation through interactions with cellulosic polymers, thereby modifying the rheological response of the system [39]. However, only DOE-4, which also contained 3% HPMC, remained physically stable during short-term elevated temperature storage. This comparison suggests that CHI-induced electrostatic structuring alone is insufficient to ensure macroscopic robustness; adequate HPMC content appears necessary to reinforce the continuous phase and promote bulk rheological structuring.

Increasing AZA concentration was generally associated with higher *η_0_* values, consistent with enhanced particle–particle and particle–polymer interactions and progressive densification of the concentrated dispersion at elevated solid loadings. A statistically significant inverse association between *η_0_* and PDI (*p* < 0.05) further indicates that increased bulk structuring contributes to improved dispersion homogeneity. Notably, the relationship between η_0_ and particle size was not strictly monotonic. While insufficient bulk structuring (low *η_0_*) was associated with broader size distributions, excessively high viscosity may restrict effective stress transmission during milling due to increased energy dissipation within the continuous phase, thereby limiting further particle breakage. This interpretation is consistent with previous reports highlighting the critical role of suspension viscosity in wet media milling efficiency [40]. Overall, these observations indicate the existence of a rheological window in which bulk rheological structuring is sufficient to preserve dispersion homogeneity without compromising comminution efficiency.

The rheological findings suggest that the observed differences in physical robustness are associated with differences in rheological structuring, which in turn are consistent with differences in the inferred bulk microstructural organisation of the concentrated dispersions. Optimal stability is therefore achieved not at the extremes of viscosity or surface charge, but within a coupled formulation–process window that balances comminution efficiency and structural resilience.

### 3.4. Crystal Growth and Short-Term Stability

Crystal growth in concentrated AZA nanosuspensions is governed by the interplay between particle size and storage temperature, which together determine both the initial physical stability of freshly milled formulations and their subsequent short-term stability during 10 days of storage.

#### 3.4.1. Initial Physical Stability

Microscopic evaluation performed immediately after milling revealed pronounced differences in the initial physical stability of the formulations (Table 4). All PB-milled formulations remained free of detectable crystal growth after milling, while 7 of 10 YZB-processed systems exhibited crystal growth. Nominal logistic regression identified milling regime (*p* = 0.0001) and AZA concentration (*p* = 0.0025) as significant predictors of crystal growth, with no significant lack of fit (*p* = 0.9979), indicating robust model performance.

YZB milling consistently produced smaller particle sizes than PB systems, with most YZB formulations in the sub-400 nm range. Within this range, the probability of crystal growth increased markedly, indicating that particle sizes below ~400 nm represent a critical threshold for process-induced instability. Although isolated exceptions occurred, logistic modelling showed that reduced particle size significantly increased the likelihood of initial instability. Logistic models for initial, RT, and 40 °C datasets all showed statistically significant fits (*p* ≤ 0.0004) with no evidence of lack of fit (*p* ≥ 0.79), and high discriminative performance (AUC = 0.92–0.94, area under the receiver operating characteristic curve), indicating excellent separation between stable and unstable systems.

This behaviour is consistent with dissolution-driven instability mechanisms. Smaller nanocrystals have higher curvature and increased dissolution rates, generating local supersaturation that promotes rapid reprecipitation or Ostwald ripening [41,42,43]. Thus, the higher stress intensity associated with YZB milling, while effective for size reduction, simultaneously increases the thermodynamic driving force for recrystallisation. In addition, the use of YZB beads resulted in greater temperature increases during milling (28–32 °C), which may adversely affect the stability of temperature-sensitive nanosuspensions such as AZA, whereas PB enabled milder thermal conditions (23–25 °C), reducing the risk of destabilisation due to higher AZA solubility at elevated temperatures [25].

Importantly, *η_0_* did not correlate with initial stability. Several YZB formulations lacking a defined low-shear plateau remained stable, while others with measurable bulk structuring showed rapid recrystallisation, indicating that initial instability is primarily governed by a process–size interaction rather than bulk rheological reinforcement.

#### 3.4.2. Short-Term Storage Stability

To isolate storage-induced destabilisation from process-related effects, only formulations initially free from detectable crystal growth were included in the short-term stability evaluation. After 10 days, temperature-dependent effects were observed (Table 4). At room temperature, all PB-milled systems remained stable, whereas storage at 40 °C induced crystal growth in a subset of formulations.

**Figure 3:**
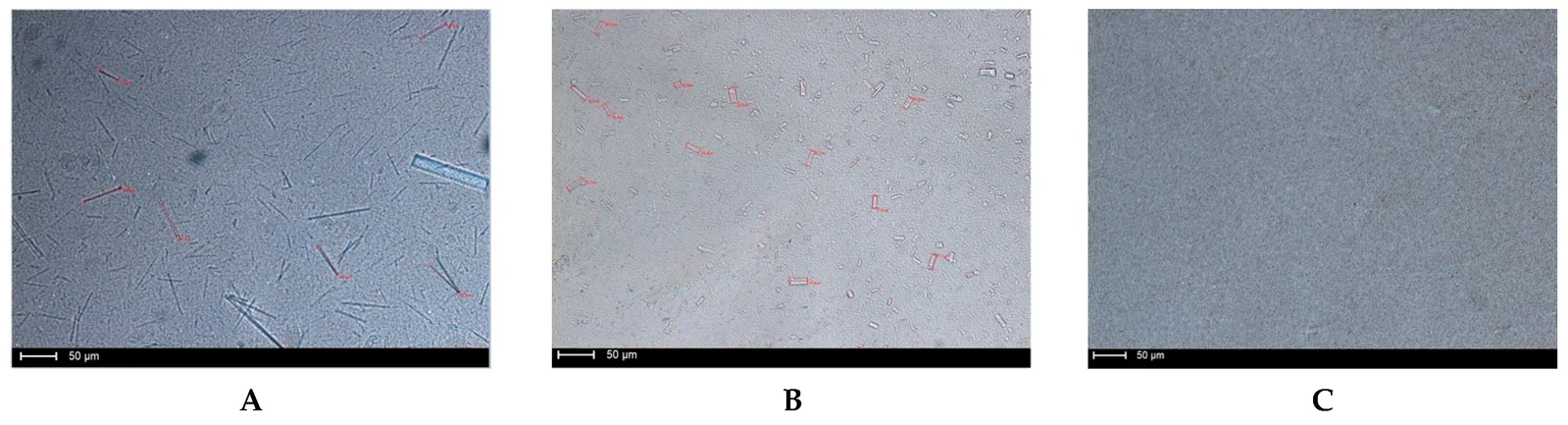
Representative optical micrographs (200× magnification) illustrate crystal growth behaviour in concentrated AZA nanosuspensions after storage for 10 days at 40 °C. (**A**) DOE-1 and (**B**) DOE-2 display pronounced needle-like recrystallisation structures, indicative of rapid dissolution–reprecipitation processes. (**C**) DOE-12 shows no visible crystalline growth, consistent with its initial physical stability. Scale bar: 50 μm.

Representative micrographs (Figure 3) show extensive needle-like crystal formation in unstable formulations (DOE-1 and DOE-2) after 10 days of storage at 40 °C, whereas no visible crystalline structures were detected in the stable formulation DOE-12. These morphological differences confirm that the observed instability is associated with rapid recrystallisation due to Ostwald ripening rather than gradual particle aggregation.

**Figure 4:**
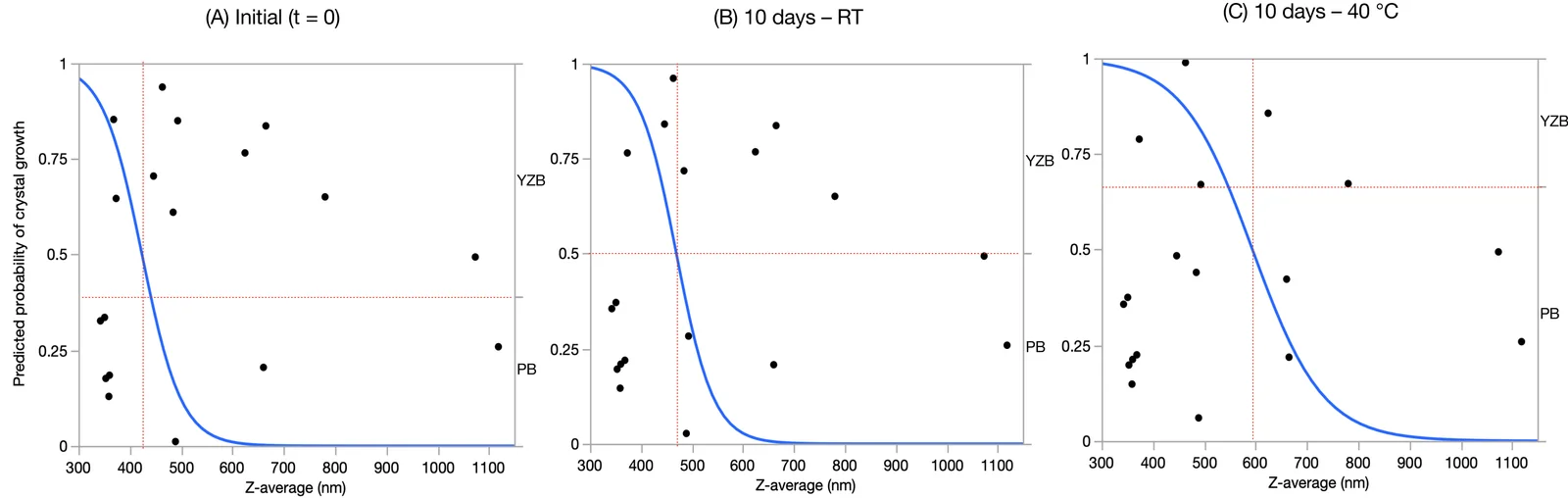
Logistic regression models describing the relationship between initial particle size (Z-average) and the probability of crystal growth in concentrated AZA nanosuspensions: (**A**) immediately after preparation (t = 0), (**B**) after 10 days at room temperature (RT), and (**C**) after 10 days at 40 °C. Vertical dashed lines indicate the estimated particle-size transition points (predicted probability = 0.5) derived from the fitted logistic regression models and should be interpreted as model-derived transition points rather than fixed physical thresholds. The secondary y-axis identifies the categorical milling regime included in the logistic regression model (YZB and PB), and the horizontal dashed lines indicate the corresponding categorical levels.

Logistic regression analysis of the initially stable formulations demonstrated a significant relationship between initial particle size and the probability of storage-induced crystal growth, with statistically significant model fits and no evidence of lack of fit. A clear size-dependent probability profile was observed (Figure 4), with the estimated particle-size transition point (predicted probability = 0.5) located at approximately 424 nm after storage at room temperature, shifting to 594 nm under accelerated conditions (40 °C).

This temperature-dependent shift indicates that larger particles become susceptible to crystal growth under accelerated storage conditions. Consistent with dissolution-driven mechanisms described by the Noyes–Whitney relationship, increased AZA solubility and diffusion kinetics at elevated temperatures provide a plausible explanation for the shift in the estimated particle-size transition towards larger particle sizes and the expanded range associated with an increased crystal growth probability [41,42,43]. These estimated transition points should not be interpreted as universal critical particle sizes but rather as system-specific characteristics reflecting the physicochemical properties of AZA, the HPMC–CHI stabilisation system, and the applied wet media milling process. Different drug substances, stabiliser combinations and processing conditions would be expected to exhibit different transition regions.

The observed storage stability could not be explained by ζ-potential alone, indicating that electrostatic contributions are insufficient to suppress recrystallisation in concentrated systems. Because CHI provides electrosteric rather than purely electrostatic stabilisation, the measured ζ-potential reflects only one component of the stabilisation mechanism. The steric contribution arising from hydrated polymer layers is not directly captured by ζ-potential measurements. Accordingly, ζ-potential should not be interpreted as the sole predictor of stability in electrosterically stabilised systems [4,44,45].

Formulations containing higher HPMC concentrations generally exhibited improved stability under accelerated conditions. Although the present data do not allow the independent contribution of HPMC to be distinguished from the concurrent effects of particle size and formulation composition, the observed behaviour is consistent with the proposed role of HPMC in reinforcing the continuous phase and increasing steric constraints on crystal growth [46,47].

Notably, changes in Z-average and PDI during storage did not consistently reflect recrystallisation detected by optical microscopy (Table 4). This apparent discrepancy arises from the different information provided by the two techniques. DLS reports an ensemble-averaged hydrodynamic particle size distribution dominated by the prevailing nanoparticle population, whereas optical microscopy directly visualises individual microcrystalline structures. This observation further supports a dissolution–recrystallisation mechanism that is not adequately captured by ensemble-averaged DLS measurements.

While optical microscopy proved suitable for identifying microscopically visible needle-like structures associated with formulation instability, complementary analytical techniques such as Raman spectroscopy, SEM or post-storage solid-state characterisation could further elucidate the structural nature of these features and the underlying recrystallisation process in future studies.

All formulations included in the storage study remained readily redispersible after vigorous shaking for approximately 10 s, with no visible signs of irreversible caking or agglomeration. These observations indicate that the detected instability was associated with crystal growth rather than irreversible sedimentation.

Overall, the short-term physical stability of concentrated AZA nanosuspensions was governed by the balance between the size- and temperature-dependent thermodynamic driving forces that promote crystal growth and the kinetic constraints imposed by the HPMC–CHI stabilisation system.

The present stability assessment was limited to a 10-day storage period and was intended as a comparative short-term screening of formulation robustness rather than a prediction of long-term storage stability. Accordingly, the observed formulation–process relationships should be interpreted within the experimental timeframe.

### 3.5. Mechanistic Formulation–Process Space and Processability Boundaries

Integration of particle size modelling, electrokinetic analysis, rheological characterisation, and stability assessment defines a coupled formulation–process space that governs the behaviour of concentrated AZA nanosuspensions. Within this space, physical robustness results from coordinated control of stress intensity during milling, interfacial charge development, and polymer-mediated bulk structuring.

The milling bead material determines the comminution regime and thus controls the extent of surface area generation. High-energy YZB milling regimes promote efficient size reduction but simultaneously increase the thermodynamic driving force for dissolution-driven recrystallisation phenomena in concentrated systems. In contrast, lower-energy PB milling regimes operate under milder stress conditions, producing larger particle sizes that may reduce the risk of recrystallisation but require adequate structural reinforcement to maintain uniform dispersion.

At the interface, CHI contributes to electrosteric stabilisation by providing a positively charged polymer layer that generates electrostatic repulsion while simultaneously contributing a steric barrier through hydrated polymer chains. However, surface charge alone does not define stability boundaries. Instead, bulk rheological organisation, as reflected in zero-shear and infinite-shear viscosity, governs particle mobility and the kinetics of crystal growth. Insufficient structuring permits particle rearrangement and growth, while excessive structuring may restrict comminution efficiency.

Accordingly, the operational window for stable, processable high-solid-content AZA nanosuspensions lies within a defined balance: particle size must be reduced sufficiently to achieve nanoscale dispersion, but not to the extent that dissolution-driven destabilisation dominates; stabiliser concentrations must provide both interfacial coverage and kinetic restriction without reducing milling efficiency. This balance defines the practical processability boundaries for high-solid-content AZA nanosuspensions.

The broadly consistent particle-size responses observed for the independently prepared replicate batches support the repeatability of particle-size control at the replicated design points. However, the larger variability observed in low-shear rheological parameters indicates that additional independent batches would be required to establish process reproducibility across the entire investigated formulation–process space. Nevertheless, although the present study was conducted at laboratory scale, wet media milling is widely recognised as a scalable pharmaceutical manufacturing technology. The formulation–process relationships identified in the present study, therefore, provide a rational basis for future process transfer and scale-up while maintaining the critical quality attributes of concentrated AZA nanosuspensions.

### 3.6. Solid-State and Morphological Characterisation of the Selected Formulations

To confirm that the observed differences in crystal growth behaviour were not attributable to solid-state transformations induced during wet media milling, selected formulations were subjected to solid-state and morphological characterisation. These analyses verified the preservation of the crystalline integrity of AZA under the investigated milling conditions and confirmed that the observed differences in physical stability could not be explained by detectable changes in the solid state of the drug.

#### 3.6.1. X-Ray Diffraction (XRD) Analysis and Solid-State Considerations

**Figure 5:**
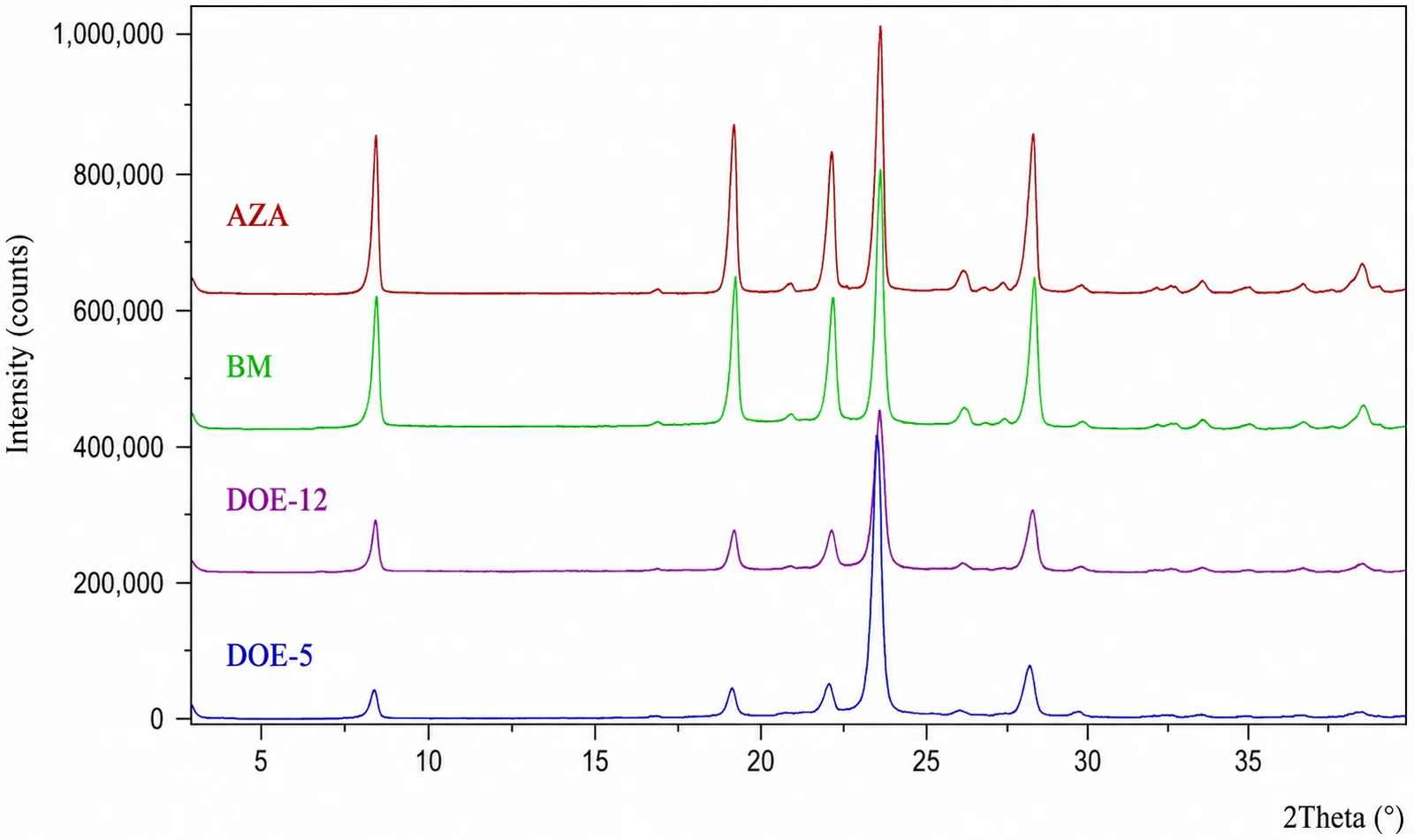
XRD patterns of pure AZA, AZA suspension before milling (BM), and composition-matched AZA nanosuspensions (20% AZA, 3.0% HPMC, 0.3% CHI) after wet media milling: DOE-12 processed under PB regime and DOE-5 processed under YZB regime.

XRD analysis was conducted on pure AZA, the suspension before milling (BM), and two composition-matched nanosuspensions (20% *w*/*w* AZA, 3.0% *w*/*w* HPMC, 0.3% *w*/*w* CHI) processed with either YZB (DOE-5) or PB (DOE-12) (Figure 5). In all samples, the characteristic diffraction peaks of the stable β-polymorphic form of AZA [48] were retained, confirming that wet media milling conditions did not induce detectable polymorphic transformation. This finding supports the interpretation that the observed differences in physical stability cannot be attributed to changes in the crystal form of AZA, but instead reflect differences in formulation composition and processing conditions.

Differences in secondary peak intensity followed the expected trend (AZA > BM > DOE-12 > DOE-5), consistent with increasing mechanical stress during milling [25]. Despite clear differences in particle size between formulations, no pronounced XRD peak broadening was observed. Furthermore, qualitative comparison of diffraction peak intensities alone does not permit discrimination between changes in crystallinity, crystallite size effects, lattice disorder, preferred orientation, or sample preparation artefacts. Nevertheless, all analysed samples retained the characteristic diffraction peaks of the β-polymorphic form of AZA, indicating preservation of crystalline identity under the investigated milling conditions.

Together, these findings support earlier observations that increasing milling intensity may introduce subtle changes in the solid-state characteristics of AZA while preserving its overall crystalline identity [25]. Within the investigated milling regimes, no detectable polymorphic transformation was observed, indicating that differences in physical stability are unlikely to originate from changes in crystal form alone.

#### 3.6.2. Scanning Electron Microscopy (SEM)

**Figure 6:**
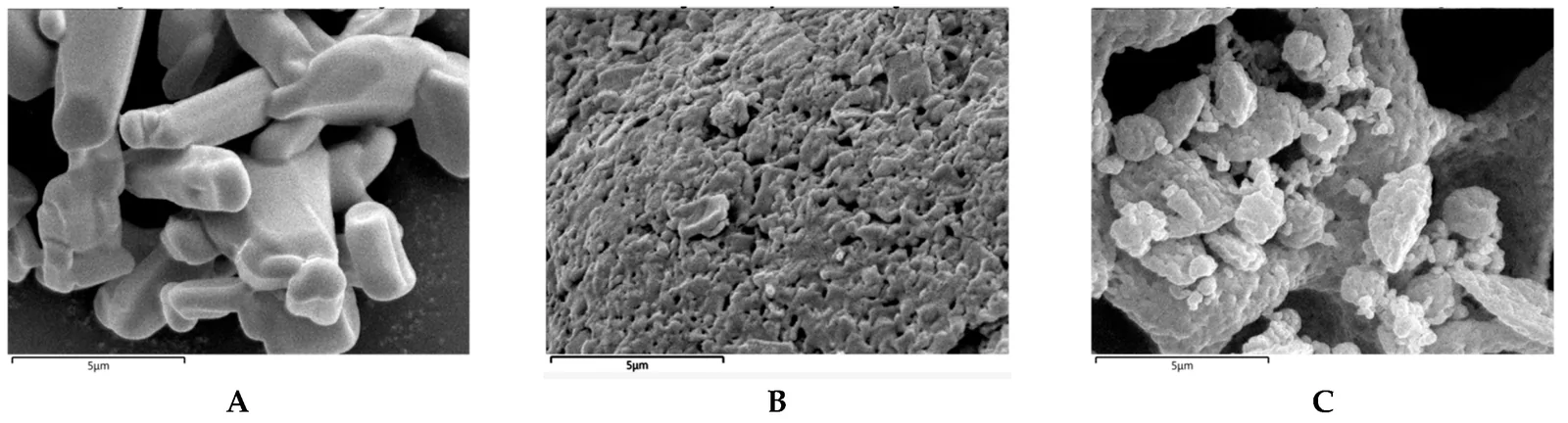
Scanning electron micrographs of azelaic acid samples at 10,000x magnification. (**A**) Pure AZA (unprocessed); (**B**) DOE-12 formulation (20% AZA, 3.0% HPMC, 0.3% CHI; milled under PB regime); (**C**) DOE-17 formulation (20% AZA, 3.0% HPMC, 0.1% CHI; milled under PB regime). Scale bar corresponds to 5 µm.

SEM was used to qualitatively examine nanocrystal morphology and spatial organisation in selected formulations with different CHI concentrations (DOE-12 and DOE-17), while maintaining identical AZA and HPMC content (Figure 6).

Representative micrographs (Figure 6A–C) show that pure AZA consists of well-defined crystalline particles, while both milled formulations display fragmented crystalline domains embedded within a continuous polymer-rich matrix. The required lyophilisation step before SEM analysis partially limited differentiation between individual nanocrystals and the surrounding polymer matrix. DOE-12 (0.3% CHI) displays more uniformly distributed crystalline domains within a denser matrix environment (Figure 6B), whereas DOE-17 (0.1% CHI) shows more closely packed crystalline regions with less apparent matrix embedding (Figure 6C).

### 3.7. In Vitro Permeation Studies

In vitro permeation studies were conducted to provide preliminary insight into the AZA transport behaviour from selected AZA-NSs across a Strat-M membrane [49,50]. Although the particle size range (400–700 nm) does not correspond to ultrafine nanocrystals, dissolution is still expected to occur relatively rapidly and is therefore unlikely to represent the primary rate-limiting step under the applied experimental conditions. Accordingly, permeation, which includes dissolution, matrix diffusion, and barrier transport, was selected as a more discriminative and biorelevant assessment of formulation performance. This approach is supported by previous reports highlighting the rapid dissolution of nanonised drug particles, the limited discriminatory power of conventional dissolution testing for these systems, and the challenges associated with separating dissolved from undissolved fractions [41]. Selected formulations (DOE-4, DOE-9, DOE-12, and DOE-17) were chosen to represent distinct regions of the investigated formulation–process space with respect to drug loading, polymer composition, and rheological behaviour.

**Figure 7:**
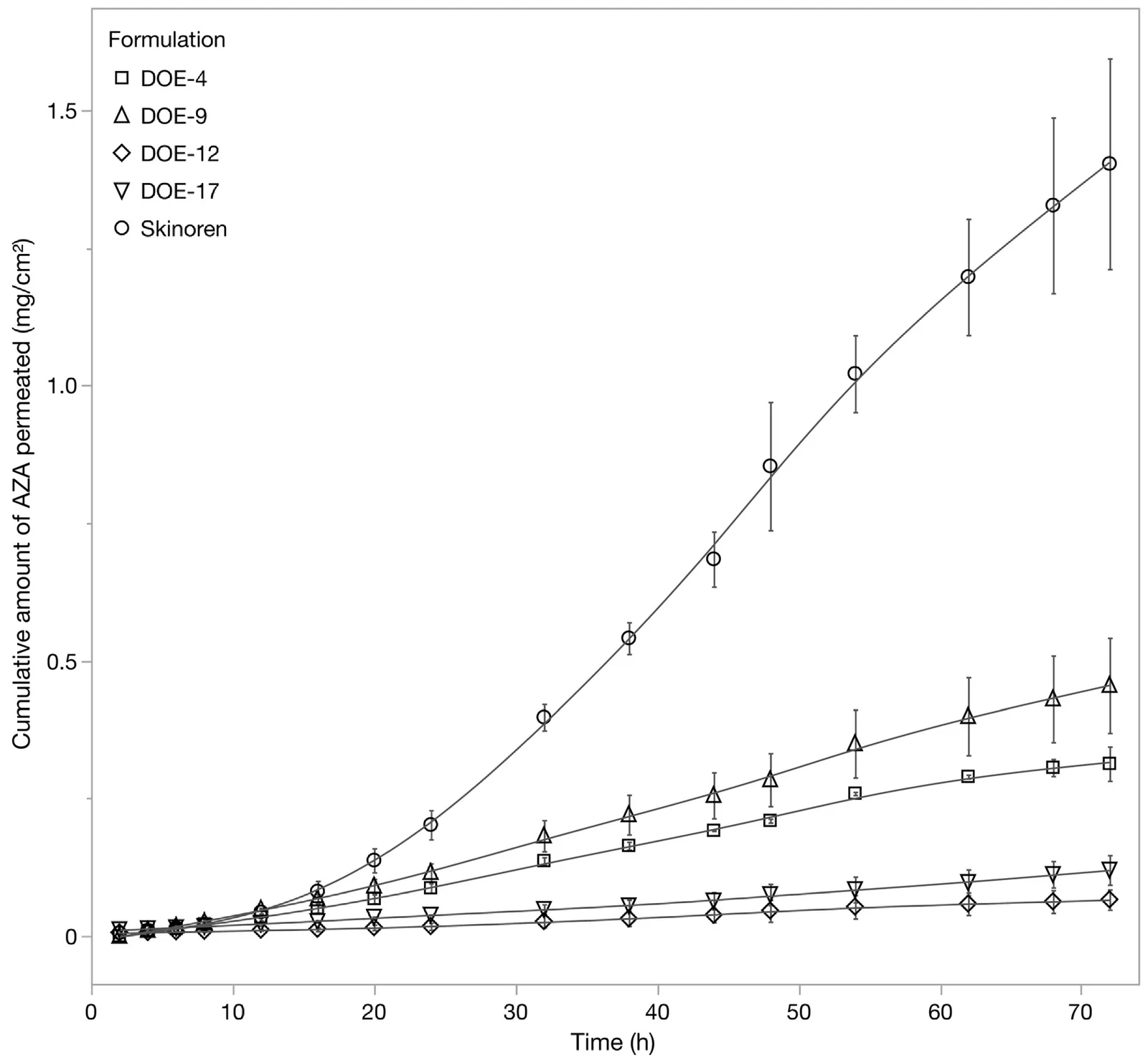
Cumulative AZA permeation across Strat-M membranes over time for Skinoren and selected AZA nanosuspensions. Data are presented as mean ± SD (n = 3). Symbols represent formulations as follows: ○ Skinoren, △ DOE-9, □ DOE-4, ▽ DOE-17, ◇ DOE-12.

Cumulative permeation profiles (Figure 7) showed markedly higher permeation for the reference formulation (Skinoren; *J* = (2.81 ± 0.64) × 10^−2^ mg·cm^−2^·h^−1^) compared with the nanosuspensions, which exhibited flux values in the 10^−3^ mg·cm^−2^·h^−1^ range (Table 5). Despite clear differences in permeation kinetics among the tested formulations, the overall extent of AZA permeation across the Strat-M membrane remained low, with cumulative amounts not exceeding approximately 1.5% for the reference product and ranging from about 0.1% to 0.8% for the DOE formulations. While these values limit direct quantitative comparison of absolute permeation efficiency, they are sufficient to enable a comparative assessment of formulation-dependent permeation trends within the investigated experimental model.

Differences between formulations were generally consistent with their rheological properties, although the present experiments do not allow the individual contributions of rheology, particle size, dissolution behaviour, and polymer–membrane interactions to be distinguished. DOE-12, which exhibited the highest zero-shear viscosity (*η_0_* ≈ 4.5 × 10^3^ mPa·s), a short relaxation time (*λ* ≈ 3.4 s), and a higher flow behaviour index (*n* ≈ 0.70), showed the lowest permeation rate, indicating a most structured and diffusion-restrictive microenvironment. In contrast, lower-viscosity systems (DOE-9 and DOE-4) exhibited higher permeation. No clear difference beyond experimental variability was observed between DOE-12 and DOE-17, both containing 20% *w*/*w* AZA.

Overall, the results indicate that HPMC–CHI-stabilised AZA nanosuspensions behave as structured vehicles in which multiple formulation characteristics, including nanocrystal–polymer interactions and the viscous microstructure, collectively contribute to reduced AZA transport across the Strat-M membrane. However, the present experiments do not allow the individual contributions of particle size, dissolution behaviour, rheological structuring and polymer–membrane interactions to be distinguished.

Because Strat-M is a synthetic membrane intended primarily for comparative permeation screening, the resulting permeation profiles should be interpreted as comparative formulation characteristics rather than as direct predictors of dermal drug delivery or clinical performance. Moreover, Strat-M does not reproduce appendageal transport pathways or drug retention within biological skin layers. Consequently, evaluation of skin retention or follicular deposition requires dedicated studies using biological skin models.

## 4. Conclusions

This study shows that in concentrated AZA nanosuspensions, stability is governed by the interplay of milling-induced stress intensity, particle size-dependent thermodynamic driving forces, interfacial charge regulation, and polymer-mediated bulk structuring, which together establish a particle size threshold below which thermodynamic instability dominates.

Nanoscale dispersions were achieved and maintained in 10–20% *w*/*w* AZA systems through controlled wet media milling combined with dual polymer stabilisation. Increased milling energy enhances comminution efficiency but simultaneously increases surface curvature and the risk of dissolution-driven recrystallisation. Reduced stress intensity produces larger particles but requires sufficient polymer-mediated structuring to preserve particle size uniformity in the dispersion. Within this coordinated stress–structure framework, CHI regulates interfacial charge, while HPMC controls rheological organisation and restricts particle mobility. Robustness results from integrated control rather than maximisation of a single parameter.

Importantly, in vitro permeation studies showed that these structured nanosuspensions act as diffusion-controlled vehicles, exhibiting lower flux but more uniform, near-linear permeation profiles compared with the reference formulation. Drug transport is governed not solely by nanocrystal size, but by the combined effects of particle size distribution, nanocrystal–polymer interactions, and the viscous microenvironment, which collectively impose resistance to diffusion and modulate release kinetics.

By integrating multivariate statistical modelling with rheological, solid-state, and permeation characterisation, this work provides a comprehensive understanding of the formulation–process relationships governing concentrated dermal AZA nanosuspensions. Within the investigated formulation–process space, the most promising formulation–process window comprised particle sizes of approximately 450–650 nm with 3.0% (*w*/*w*) HPMC and 0.1–0.3% (*w*/*w*) CHI, produced under the lower-stress PB milling regime. These conditions provided the most favourable balance of milling efficiency, short-term physical stability, and rheological structuring, while remaining specific to the investigated AZA–HPMC–CHI nanosuspension system.

## Figures and Tables

**Table 1:** Formulation and process factors investigated in the Design of Experiments.

**Formulation Factors**	**Levels**			
	**Low (–1)**	**Medium (0)**		**High (+1)**
X1: AZA (% *w*/*w*)	10	15		20
X2: HPMC (% *w*/*w*)	1.50	2.25		3.00
X3: CHI (% *w*/*w*)	0.1	0.2		0.3
**Process Factors**	**Levels**			
Milling regime	**YZB** (0.1–0.2 mm beads; 60 min)		**PB** (0.45–0.55 mm beads; 240 min)	

**Table 2:** Experimental design matrix and corresponding physicochemical responses of azelaic acid nanosuspensions, including particle size (Z-average), polydispersity index (PDI), zeta potential (ζ), and rheological parameters obtained from Cross model fitting.

Run	AZA (% *w*/*w*)	CHI (% *w*/*w*)	HPMC (% *w*/*w*)	Milling Regime	Z-Average (nm) ± SD	PDI ± SD	ζ (mV) ± SD	Rheological Parameters ^1^			
								$\eta_{0}$ (mPa·s) ± SD	$\eta_{\infty }$ (mPa·s) ± SD	λ	n (-)
DOE -1	20	0.3	1.50	YZB	342.7 ± 2.57	0.186 ± 0.016	38.5 ± 0.624	1686 ± 270	10.6 ± 2.70	6.4 ± 3.4	0.57 ± 0.04
DOE-2	20	0.1	3.00	YZB	360.3 ± 2.23	0.207 ± 0.020	25.5 ± 0.850	45,462 ± 8562	24.5 ± 3.0	33.3 ± 1	0.87 ± 0.02
DOE-3	20	0.1	3.00	YZB	373.2 ± 0.64	0.220 ± 0.050	23.5 ± 2.340	4905 ± 321	24.0 ± 3.40	3.0 ± 0.52	0.85 ± 0.03
DOE-4	10	0.3	3.00	YZB	624.3 ± 17.8	0.344 ± 0.064	46.4 ± 0.777	*	25.7 ± 1.30	222 ± 38.3	0.5 ± 0.02
DOE-5	20	0.3	3.00	YZB	350.8 ± 2.96	0.219 ± 0.021	40.8 ± 1.100	7975.8 ± 573	35.5 ± 2.50	10.8 ± 2	0.67 ± 0.02
DOE-6	20	0.1	1.50	YZB	359.1 ± 1.82	0.195 ± 0.013	18.5 ± 0.265	5937 ± 908	14.2 ± 4.00	5.4 ± 1.8	0.94 ± 0.07
DOE-7	15	0.2	2.25	YZB	353.1 ± 2.54	0.230 ± 0.008	35.9 ± 0.751	1175 ± 94.6	11.6 ± 1.00	7.9 ± 2	0.55 ± 0.02
DOE-8	10	0.3	1.50	YZB	488.9 ± 8.11	0.273 ± 0.002	47.9 ± 0.961	*	9.6 ± 0.64	371 ± 120	0.54 ± 0.02
DOE-9	10	0.1	1.50	YZB	493.2 ± 5.96	0.271 ± 0.005	33.8 ± 0.608	*	3.4 ± 0.53	1610 ± 661	0.33 ± 0.02
DOE-10	10	0.1	3.00	YZB	463.3 ± 6.64	0.237 ± 0.005	38.8 ± 0.321	*	13.3 ± 0.94	634 ± 330	0.44 ± 0.04
DOE-11	15	0.2	2.25	YZB	368.4 ± 3.67	0.206 ± 0.013	31.2 ±1.380	*	12.8 ± 1.20	366 ± 27	0.66 ± 0.01
DOE-12	20	0.3	3.00	PB	660.3 ± 4.37	0.235 ± 0.015	43.4 ± 0.300	4487 ± 391	32.0 ± 3.40	3.4 ± 0.8	0.70 ± 0.02
DOE-13	10	0.3	1.50	PB	1118.0 ± 18.00	0.393 ± 0.009	43.4 ±0.351	114 ± 8.40	12.8 ± 0.70	0.92 ± 0.25	0.73 ± 0.07
DOE-14	20	0.2	1.50	PB	484.3 ± 5.63	0.301 ± 0.027	38.6 ± 0.458	*	21.4 ± 5.80	58.9 ± 4.4	0.88 ± 0.02
DOE-15	10	0.2	3.00	PB	1073.0 ± 40.60	0.367 ± 0.003	45.8 ± 0.721	*	26.7 ± 1.70	3140 ± 733	0.53 ± 0.02
DOE-16	10	0.1	1.50	PB	665.0 ± 14.90	0.327 ± 0.014	31.6 ± 0.458	68 ± 17	6.0 ± 0.20	5.9 ± 4.8	0.59 ± 0.05
DOE-17	20	0.1	3.00	PB	446.1 ± 4.11	0.227 ± 0.010	26.7 ± 0.577	2171 ± 188	25.4 ± 2.30	5 ± 1.4	0.64 ± 0.02
DOE-18	15	0.2	2.25	PB	780.3 ± 17.60	0.356 ± 0.047	31.3 ± 0.551	*	15.0 ± 1.00	16.2 ± 2	0.51 ± 0.03

^1^ Best-fitting parameters according to the Cross model. An asterisk (*) indicates that zero-shear viscosity (*η_0_*) could not be determined with statistical reliability due to the absence of a defined low-shear plateau.

**Table 3:** Reduced regression models and statistical parameters describing the influence of formulation and process variables on particle characteristics and rheological parameters of wet-milled AZA nanosuspensions.

Response	Reduced Regression Model *	R^2^	R^2^_adj_	Model *p*-Value	LOF *p*-Value
Z-average (nm)	Z_avg_ = 756.08 − 30.16·AZA + 904.04·CHI + 43.70·HPMC + 155.75·D_1_ − 13.83·(AZA×D_1_) − 68.30·(AZA×CHI) + 744.99·(CHI×D_1_)	0.97	0.94	<0.001	0.0235
PDI	PDI = 0.383 − 0.0083·AZA + 0.137·CHI − 0.004·HPMC + 0.0377·D_1_ − 0.0294·(AZA×CHI) − 0.0198·(HPMC×D_1_)	0.88	0.81	0.0001	0.1824
Zeta potential, *ζ* (mV)	*ζ* = 30.17 − 0.87·AZA + 72.89·CHI + 2.08·HPMC + 0.86·D_1_	0.84	0.79	<0.001	0.3164
Infinite-shear viscosity, *η∞* (mPa·s)	*η_∞_* = −31.60 + 1.0365·AZA + 43.0367·CHI + 9.1397·HPMC + 0.4880·AZA^2^ − 3.2911·(AZA×CHI) − 810·CHI^2^ + 0.3421·(AZA×HPMC) + 31.3929·(CHI×HPMC)	0.98	0.96	<0.0001	0.2979
Flow behaviour index, *n* (–)	*n* = 0.37565 + 0.0255·AZA − 0.17128·CHI − 0.04043·HPMC + 0.02107·D_1_ − 0.16193·(AZA×CHI)	0.74	0.57	0.0076	0.2001

* Reduced polynomial models were obtained using DoE analysis. Regression coefficients are expressed in coded factor units. Model adequacy was evaluated by the coefficient of determination (R^2^), adjusted coefficient of determination (R^2^adj), predicted coefficient of determination (R^2^pred), overall model significance (Model *p*-value), lack-of-fit testing (LOF), and residual diagnostics. AZA = azelaic acid concentration (% *w*/*w*); CHI = chitosan concentration (% *w*/*w*); HPMC = hydroxypropyl methylcellulose concentration (% *w*/*w*); D_1_ = milling regime (categorical factor: YZB or PB); *η∞* = infinite-shear viscosity derived from the Cross model; *n* = flow behaviour index derived from the Cross model. Only statistically significant terms (*p* < 0.05) were retained in the reduced models unless hierarchical structure required term preservation.

**Table 4:** Initial and short-term physical stability of concentrated AZA nanosuspensions assessed by microscopic detection of crystal growth.

Run	Milling Media	Initial Stability	Stability After 10 Days at 40 °C			Stability After 10 Days at RT		
		Crystal Growth ^1^	Δ Z-Average (nm)	Δ Zeta Potential (mV)	Crystal Growth ^1^	Δ Z-Average (nm)	Δ Zeta Potential (mV)	Crystal Growth ^1^
DOE-1	YZB	1	ND	ND	ND	ND	ND	ND
DOE-2	YZB	1	ND	ND	ND	ND	ND	ND
DOE-3	YZB	1	ND	ND	ND	ND	ND	ND
DOE-4	YZB	0	−94.90	−0.50	0	−92.60	−3.10	0
DOE-5	YZB	1	ND	ND	ND	ND	ND	ND
DOE-6	YZB	1	ND	ND	ND	ND	ND	ND
DOE-7	YZB	1	ND	ND	ND	ND	ND	ND
DOE-8	YZB	1	ND	ND	ND	ND	ND	ND
DOE-9	YZB	0	231.50	−6.60	1	−60.20	−8.20	1
DOE-10	YZB	0	44.50	−9.20	0	34.40	−9.10	0
DOE-11	YZB	0	18.30	3.80	1	20.90	−1.30	1
DOE-12	PB	0	9.30	−3.70	0	−9.80	3.50	0
DOE-13	PB	0	−118.30	2.70	0	−199.4	−3.80	0
DOE-14	PB	0	−56.20	0.40	1	−95.90	0.00	0
DOE-15	PB	0	−44.00	−11.90	0	−81.10	−5.90	0
DOE-16	PB	0	0.40	−4.40	1	−42.10	−4.10	0
DOE-17	PB	0	95.20	−4.20	1	87.50	−3.30	0
DOE-18	PB	0	119.60	8.20	0	347.70	6.40	0

^1^ Crystal growth was assessed by optical microscopy immediately after preparation and after 10 days of storage at RT and 40 °C. Samples exhibiting microscopically visible needle-like structures consistent with crystal growth (>2 μm) were assigned a value of “1”, whereas samples without detectable crystalline structures were assigned “0”. Formulations exhibiting structures consistent with crystal growth immediately after preparation were excluded from short-storage analyses and regression modelling of storage-induced instability. Δ Z-average and Δ Zeta potential values represent the difference in mean particle size and zeta potential relative to initial measurements. ND = not determined because of initial instability; ND entries are shown in gray for visual distinction.

**Table 5:** Permeation parameters of azelaic acid across Strat-M membranes determined from steady-state permeation profiles (mean ± SD, n = 3).

Formulation	Flux *J* (mg·cm^−2^·h^−1^)	*P*_app_ (cm·s^−1^)
Skinoren	(2.81 ± 0.64) ×10^−2^	(5.98 ± 2.77) ×10^−8^
DOE-9	(6.37 ± 2.81) ×10^−3^	(1.87 ± 0.88) ×10^−8^
DOE-4	(5.34 ± 0.31) ×10^−3^	(1.62 ± 0.13) ×10^−8^
DOE-17	(1.81 ± 0.45) ×10^−3^	(2.26 ± 0.53) ×10^−9^
DOE-12	(1.01 ± 0.23) ×10^−3^	(1.29 ± 0.30) ×10^−9^

Flux (*J*) was determined from the slope of the linear portion of the cumulative permeation profile. The apparent permeability coefficient (*P*_app_) was calculated as *P*_app_ = *J*/*C*_d_, where *C*_d_ represents the nominal total AZA concentration in the donor formulation.

## Data Availability

All data generated or analysed during this study are included in this article.
